# Clinically Relevant Characterization and Comparison of Ryaltris and Other Anti-Allergic Nasal Sprays

**DOI:** 10.3390/pharmaceutics16080989

**Published:** 2024-07-26

**Authors:** Virginia Patterlini, Fabiola Guareschi, Davide D’Angelo, Simone Baldini, Suada Meto, Dalia Mostafa Kamal, Paolo Fabrizzi, Francesca Buttini, Ralph Mösges, Fabio Sonvico

**Affiliations:** 1 Dipartimento di Scienze Degli Alimenti e del Farmaco, Università di Parma, Parco Area delle Scienze 27/A, 43124 Parma, Italy; virginia.patterlini@unipr.it (V.P.); fabiola.guareschi@unipr.it (F.G.); davide.dangelo@unipr.it (D.D.); francesca.buttini@unipr.it (F.B.); 2 Menarini Group, Via Sette Santi 1-3, 50131 Florence, Italy; sbaldini@menarini.it (S.B.); smeto@menarini.it (S.M.); pfabrizzi@menarini.it (P.F.); 3 Biopharmanet-TEC, Università di Parma, Parco Area delle Scienze 27/A, 43124 Parma, Italy; 4 Institute of Medical Statistics and Computational Biology (IMSB), Medical Faculty, University at Cologne, 50923 Cologne, Germany; ralph@moesges.de

**Keywords:** nasal drug delivery, corticosteroids, dissolution test for inhalation products, nasal cast deposition

## Abstract

The deposition, residence time, and dissolution profile of nasal suspensions containing corticosteroids play a key role in their in vivo efficacy after administration. However, the conventional methods available to characterize nasal products appear to be unsuitable to exhaustively cover these aspects. The work aims to investigate technological aspects of Ryaltris (mometasone furoate and olopatadine hydrochloride nasal spray) compared to other commercial anti-allergic nasal products, namely, Dymista (azelastine hydrochloride and fluticasone propionate), Nasonex (mometasone furoate), and Avamys (fluticasone furoate). Innovative characterization methods were combined with more traditional approaches to investigate the anti-allergic nasal sprays. These methods applied together allowed to differentiate between the different products and provided a clear picture of the nasal product behavior in terms of drug dissolution and deposition. In particular, the dissolution tests were performed exploiting the Respicell^®^ apparatus, an innovative technique that allows for the investigation of inhalation products. Then, formulation viscosities were considered along with a formulation flow test on an inclined plane. Finally, the intranasal deposition profile of the commercial formulations was determined using a silicon nasal cast. The results highlight in vitro significant differences in terms of viscosity as well as dissolution rate of the nasal products, with Ryaltris showing a higher viscosity and lower flow compared to other products, which, along with a corticosteroid faster dissolution rate than Dymista, suggest a potential advantage in terms of clinical behavior.

## 1. Introduction

In recent years a number of nasal products have been developed and commercialized, with the aim of treating several local and systemic diseases [1,2]. In particular, nasal products are often used to treat nasal seasonal or perennial rhinitis. Nasal symptoms severely affect the quality of life of patients, with allergic rhinitis being responsible, in the United States alone, for about 800,000 days off work and 825,000 days away from school, and for a decrease in productivity estimated at 4,250,000 days per year [3]. The nasal products approved for the local treatment of seasonal allergic or perennial rhinitis are often formulated as suspensions containing corticosteroids or solutions of antihistaminic drugs, even if in some cases the combination of the two therapeutic classes is proposed, which becomes particularly relevant for the treatment of allergic rhinitis [4]. Powder formulations for nasal delivery are comparatively rarer in the market [5,6]. It is well established that the efficacy of these nasal products is based on drug solubility, stability, deposition in the nasal cavity, residence time (limited by formulation elimination due to mucociliary clearance), and the effect of excipients that altogether ultimately affect local absorption and determine the behavior of the formulations in vivo [7,8,9]. Pharmacopoeial tests and characterizations prescribed by the guidance issued by the FDA and EMA, such as uniformity of delivered dose, uniformity of delivered mass, and number of deliveries per container, although essential, seem to provide a technically accurate but incomplete evaluation of the products, lacking tests relevant for the product’s in vivo behavior [10]. In the last few years, many pharmaceutical scientists have devised for both inhalation and nasal products innovative tests to investigate the interplay between formulation spray/aerosolization, deposition, drug dissolution, and bioavailability, in order to allow better in vitro characterization and potentially a more accurate prediction of in vivo efficacy of such products [11,12,13]. The need to carry out relevant dissolution tests for products delivered to the airways with low solubility and/or high dosing has been debated over the years [14,15]. Of note, dissolution profiles have been proposed as an important tool for in vitro characterization and discrimination of inhaled products, providing a tool for quality control, for the comparison of different formulation strategies or even to compare originators with generic drugs [16,17]. In particular, RespiCell^®^, a new dissolution apparatus based on studying the dissolution at the liquid interface, could be helpful in correlating in vitro dissolution kinetics of poorly soluble products deposited in the airways with pharmacokinetics and pharmacodynamics data. The RespiCell apparatus has been shown to be useful in the characterization of inhalation products, but has not yet been applied to the systematic characterization of nasal products. At the same time, the study of spray deposition using anatomically correct models of the nasal cavity appears critical for the characterization of nasal products since it has been shown that nasal drug deposition significantly impacts the efficacy of such treatments [18]. The technique consists of using nasal casts and reproductions to study nasal product deposition in specific regions of interest in the nasal cavity (nostrils, vestibule, turbinates, olfactory region, and nasopharynx) [10]. Nasal casts can also be used to evaluate the correct and incorrect angle of administration of the nasal formulation to investigate how deposition can differ when the device is used incorrectly [11]. In the investigation of nasal formulations, even the evaluation of the adhesion has been considered quite important, since it is linked to the residence time of the product in the nasal cavity and to its absorption [12]. A method that has been employed in many published studies includes an inclined plane of 45° and a simulated mucus, where the formulation is applied and then subjected to a controlled gravitational force, in order to investigate its adhesion properties [13].

The aim of the research was to combine the above-mentioned methods focusing on dissolution, deposition, and adhesion for the characterization of the technological aspects which determine the clinical performance of Ryaltris (mometasone furoate and olopatadine hydrochloride) nasal spray in comparison to other commercial nasal products indicated for allergic rhinitis, namely, Dymista (azelastine hydrochloride and fluticasone propionate), Nasonex (mometasone furoate), and Avamys (fluticasone furoate). In particular, for these nasal sprays containing poorly soluble corticosteroids, it was interesting to clarify if using the Respicell apparatus was possible to differentiate their dissolution kinetics, ultimately to provide some further light on their respective behaviors in vivo. The results obtained were correlated to other properties studied, such as adhesion and deposition in the nasal cavity, in order to provide an all-round characterization about the products under investigation.

The research presents a significant contribution to the field by elucidating the key factors necessary for a thorough characterization and evaluation of nasal products. In particular, the research proposes innovative testing methods able to gain deeper insights into critical aspects of nasal sprays and ultimately predict their in vivo performance.

## 2. Materials and Methods

### 2.1. Materials

Four commercial nasal products were purchased by a local pharmacy (Farmacia S. Lazzaro, Parma, Italy) and are listed in Table 1. The images of the nasal products and physico-chemical properties of the active ingredients contained in the nasal products are presented in Figure S1 and Table S1, respectively (see Supplementary Materials).

Other materials employed for analytical purposes were sodium dihydrogen phosphate monohydrate and sodium hydroxide (Sigma Aldrich, Milan, Italy), potassium dihydrogen phosphate (A.C.E.F S.p.A., Fiorenzuola d’Arda, Italy), ortophosphoric acid 85% (VWR International, Milan, Italy), and triethylamine (Carlo Erba Reagents, Milan, Italy). Dodecyl sulfate sodium salt was provided by Acros Organics B.V.B.A (Geel, Belgium). Analytical standards of the active pharmaceutical ingredients (APIs) contained in the nasal commercial products, i.e., azelastine hydrochloride, fluticasone furoate, fluticasone propionate, mometasone furoate, and olopatidine hydrochloride, were all purchased from Sigma Aldrich (Milan, Italy).

All solvents, such as acetonitrile and methanol were of analytical grade.

Ultrapure water was obtained by reverse osmosis using a MilliQ apparatus (Merck Millipore, Molsheim, France).

### 2.2. Methods

#### 2.2.1. Viscosity

The viscosity of the products under study was performed using a controlled stress rheometer, HAAKE RheoStress 1 (Thermo Fisher Scientific Inc., Karlsruhe, Germany). The instrument supports the software HAAKE Rheowin (version 4.88.0002) and it employs a 4th generation air-bearing and digital signal processor (DSP) technology. The analyses were carried out using a rheometer with a plate/cone geometry of C35/2° Ti, the temperature was set at 25 °C, and the measurement gap at 0.106.

For the measurements, 1 mL of each suspension was placed on the stationary surface of the rheometer. The rotation of the cone was set at 1.5 rpm and maintained constant for 300 s. Five measurements, in the span of five minutes (one measurement per minute), were taken for each sample. The first two measurements for each analysis were discarded at the beginning of the process where the instrument takes time to reach the desired rotation.

#### 2.2.2. Formulation Flow/Dripping Test on a Coated Inclined Plane

Adhesive properties for the four products, Avamys, Dymista, Nasonex, and Ryaltris, were investigated observing the flow of the formulations on an inclined plane coated with a semisolid material, roughly simulating the mucus layer present on the mucosal surfaces. For the experiments a Fluoropolymer Coated Release Liner (No. 1022 Scotchpak film, 3M, Maplewood, MN, USA) with the dimension of 10 × 15 cm was employed. It was coated with a layer of Sargel^®^, a water-finding paste (calcium oxide, silicon oxide, phenolphthalein, polyethylene oxide, polypropylene glycol; Arkema, Exton, PA, USA) that had been spread with a settled thickness of 0.3 mm on the liner thanks to a film-casting knife (BYK, Wessel, Germany). The liner was placed on a glass slab, as support; then with a pipette, 50 µL of each formulation was deposed on the Sargel^®^ paste coating the fluorinated liner. Right after the deposition, the glass slab and therefore, the liner, were placed on an inclined plane with a 45° angle to simulate the inclination of the nasal cavity. At the end of the run, one minute after the application, images were taken for each formulation using a digital camera (Sony α 5100, Sony, Tokyo, Japan; 24.3 megapixels APS-C sensor). The length of the flowing-off distance for each formulation was calculated thanks to ImageJ software (version 1.54j, https://imagej.net/ij/, accessed on 22 July 2024) and the conversion from pixels to cm was obtained thanks to a graduated scale placed on the glass slab.

#### 2.2.3. Droplet Size Distribution

The investigation of particle size distribution was conducted with a SprayTEC laser diffraction instrument (Malvern Panalytical Ltd., Malvern, UK).

The SprayTEC instrument is an apparatus equipped with a 4 mW Helium-Neon laser that was set with a wavelength of 632.8 nm, and a receiver module equipped with a 300 mm focal lens that is suited to detect particles in the range between 0.1 µm and 900.0 µm.

All four commercial products, Ryaltris, Dymista, Nasonex and Avamys, were subjected to the analysis of spray droplet size distribution.

For each product, three devices were employed (*n* = 3) and they were analyzed at the beginning of life to evaluate the particle size distribution of each product. Every product was subjected to shaking and priming as stated in the leaflet. Each device was positioned on a polystyrene support with a 45° angle and secured with a metal clamp. The distance between the opening in the spray nozzle and the laser beam was set at 5 cm and the distance between the device and the detector window was set at 15 cm. Directly in front of the device, a fume extractor device, Arm Evac 200 (Pace Europe, Milton Keynes, UK) was positioned to collect the emitted spray and avoid particle recounting.

For data processing with the SprayTEC software (version 3.20, Malvern Panalytical Ltd., Malvern, UK), mathematical methods applied parameters such as refractive index value of the particles, density, and dispersant material refractive index were chosen as follows: the particle refractive index value was the water’s with a value of 1.33, the density was set at 1 g/cm^3^, and air was chosen as the dispersant with a refractive index of 1.

After switching on the apparatus, 6 spray activations were recorded for each device and only the portion of the measurement corresponding to the full-developed spray was analyzed.

#### 2.2.4. Deposition in a Nasal Cast

The analysis of the deposition in the nasal cast was performed on all formulations, examining in which part of the nose the formulation settles if the device was positioned with an angle of 45°, simulating the correct insertion of the device in the nostril. The silicone nasal cavity model used (Model LM-005, Koken Ltd., Tokyo, Japan) is formed by two parts, corresponding to the left and right side of the nasal cavity. Only the left side of the nasal cast was employed for the experiment of deposition of nasal products tested. A water-finding paste (Sargel^®^, Arkema, Exton, PA, USA) was spread in the whole cast to highlight the deposition of the nasal product, by changing color to bright purple when in contact with the aqueous formulation. The devices were inserted to a depth of 1 cm in the nostril with an inclination of 45°, to simulate the correct position, while Ryaltris was subjected to more tests, simulating two incorrect insertions, with inclinations of 30° and 60°, in order to assess the formulations’ deposition and observe how a non-optimal insertion angle could change the product deposition, potentially affecting the nasal spray efficacy. A digital camera (Sony α 5100, Sony, Tokyo, Japan; 24.3 megapixels APS-C sensor) was used to take the images and the parameters set for image acquisition were 1/250, F 4.5, ISO 250.

For each analysis, one minute elapsed between the actuation of the device and the imaging. The pictures were analyzed by the software ImageJ (U.S. National Institute of Health, Bethesda, MD, USA) to visualize the deposition area and region of interest. For this purpose, the nasal cavity was divided into three regions of interest (ROI): the vestibule, the middle–upper turbinate, and the lower turbinate (see Supplementary Materials, Figure S2). Images taken a minute after spraying were transformed to an 8-bit color image, and during analysis, the number of pixels was converted into mm^2^ using a graduated scale placed close to the nasal cast. For all of the captured images, the threshold-level range was fixed from 0 to 109.

#### 2.2.5. HPLC Methods for the Quantitation of Active Substances

The analytical quantification of the APIs was carried out by high-performance liquid chromatography (HPLC). The analyses were performed on a Shimadzu HPLC chromatographic system (Shimadzu, Kyoto, Japan), composed of an UV detector, an auto-sampler, and two pumps. For the detector signal processing, a Shimadzu LabSolutions multi LC-PDA software (version 5.97 SPI, Shimadzu, Kyoto, Japan) was used. The details of each HPLC method for the APIs are reported in Table 2.

The validation of each method (linearity, LOD, LOQ) is reported in the Supplementary Materials (Figures S3–S5). The analytical methods used for azelastine HCl and olopatadine HCl HPLC are reported as well (Figures S6 and S7).

#### 2.2.6. Dissolution Studies of the Nasal Formulations

In vitro dissolution tests were performed using the RespiCell dissolution apparatus (EU Design registration N006649570-0001, manufactured by DISA s.p.a, Milan, Italy; see Supplementary Materials, Figure S8) [19]. It is a vertical dissolution apparatus, composed of two parts, a donor chamber and an acceptor chamber. The latter chamber is filled with 180 mL of dissolution medium and has a sampling side arm of 10 cm length. The two chambers were separated by a polycarbonate membrane (Nucleopore^TM^, 0.4 µm pore size, diameter 90 mm, Whatman, Little Chalfont, UK) which allows direct contact of the formulation with the dissolution medium. The available surface area for dissolution and diffusion is 30.2 cm^2^. The dissolution apparatus is jacketed and was connected to a heating thermostat (Eco Silver E4, Lauda, Assago, Italy) set at 37 ± 0.5 °C.

The dissolution medium used to solubilize the water-insoluble corticosteroid APIs was composed by a phosphate-buffer saline (PBS) with an addition of sodium dodecyl sulphate (SDS) 1.5% *w*/*v*, in order to respect the sink condition (final concentration of API below 20% of its solubility). For each product analyzed, a volume equal to 30 actuations was deposited on the filter. At predetermined time points, 1 mL samples were withdrawn by a side arm of the cell and analyzed by HPLC according to the methods presented in Section 2.2.5. The dissolution compartment was refilled with 1 mL of pre-heated fresh dissolution medium. At the end of the experiment, the filter was collected and washed with 15 mL of collection solvent, in order to quantify the amount of drug not dissolved. The experiment was performed in triplicate for each product. The total amount of the drug for the experiment was calculated considering the drug dissolved plus the amount of drug found in the filter.

In all the experiments, the total amount of drug recovered at the end of the HPLC analysis was always above 85% of the drug deposited.

#### 2.2.7. Particle Size Distribution and Particle Morphology Analysis

The analysis of the particle size distribution and the particle morphology were performed using a Morphologi 4-ID equipment from Malvern Panalytical (Alfatest Lab, Milan, Italy). For each product, an aliquot of 1 mL was obtained collecting several upright-oriented actuations of the nasal spray. Then, 1:10 sample dilution was performed in purified water. These latter and the undiluted samples were first analyzed to find the best condition between excipient dissolution and particle count. With this aim, 10 µL of each suspension was used to prepare a slide and was immediately analyzed using the parameters listed below. The other two analytical repetitions were performed with the 1:10 dilutions that resulted in the best choice. The parameters set were the magnification optic of 20 × (1.5–130 µm), the diascopic illumination (70% intensity), a threshold of 0–140, and a measured area of 200 mm^2^. The following filters were applied to exclude possible aggregate particles and/or air bubbles: circularity > 0.950, convexity < 0.850, and solidity < 0.850.

For each sample, three measurements were performed, and for each measurement at least 50,000 particles were counted and individually measured in terms of projected area circular equivalent diameter. For each sample, the Dn10, Dn50, and Dn90 were calculated, corresponding to the size under which fall the 10, 50, and 90% of particles measured in the number-based distribution.

#### 2.2.8. Statistics

All results are reported as mean value and standard deviation of at least 3 replicates, if not stated otherwise. All statistics analyses were performed using Prism Software Version 8.0a (Prism, Version 8.0a, GraphPad Software Inc., La Jolla, CA, USA). Data dispersion was verified using one-way ANOVA with post hoc Tukey statistic for multiple comparisons, considering significant differences with * *p* < 0.05, ** *p* < 0.01, *** *p* < 0.001 and **** *p* < 0.0001 (Prism, Version 10.0a, GraphPad Software Inc., La Jolla, CA, USA).

The analysis of the dissolution profiles of the different drugs over time were conducted using the difference (*f*_1_) and similarity (*f*_2_) factors [20]. The percent difference between the two dissolution profiles at each time point is calculated using *f*_1_, which also measures the relative inaccuracy between the two profiles, according to Equation (1):

(1) $$f_{1}=\left(\frac{\sum_{t=1}^{n}\left|R_{t}-T_{t}\right|}{\sum_{t=1}^{n}R_{t}}\right)\;\times \;100$$

There are no significant differences in the dissolution profiles when the *f*_1_ value is lower than 15 (0–15).

Conversely, the similarity factor (*f*_2_) is a logarithmic transformation of the sum-squared error of differences between the tested products and was calculated using Equation (2):

(2) $$f_{2}=50\;\times \;\log\left[\frac{100}{\sqrt{1\;+\frac{\sum_{t=1}^{n}{\left(R_{t}-T_{t}\right)}^{2}}{n}}}\right]$$

Two dissolution profiles are considered equivalent when the *f*_2_ value is higher than 50 (50–100).

Where *n* indicates the number of time points, at time *t*, $R_{t}$ represents the mean dissolution value for the reference product, and $T_{t}$ represents the mean dissolution value for the test product. The evaluation of the similarity factor is based on the following requirements: for each formulation, no more than one mean value should exceed 85% of the dissolved drug, and a minimum of three of the same time points (with zero excluded) must be taken into account for both products. Additionally, for the initial time point and the other time points under consideration, the relative standard deviation (coefficient of variation) should be less than 20% and 10%, respectively.

## 3. Results

### 3.1. Formulation-Related Aspects

#### 3.1.1. Viscosity

**Figure 1:**
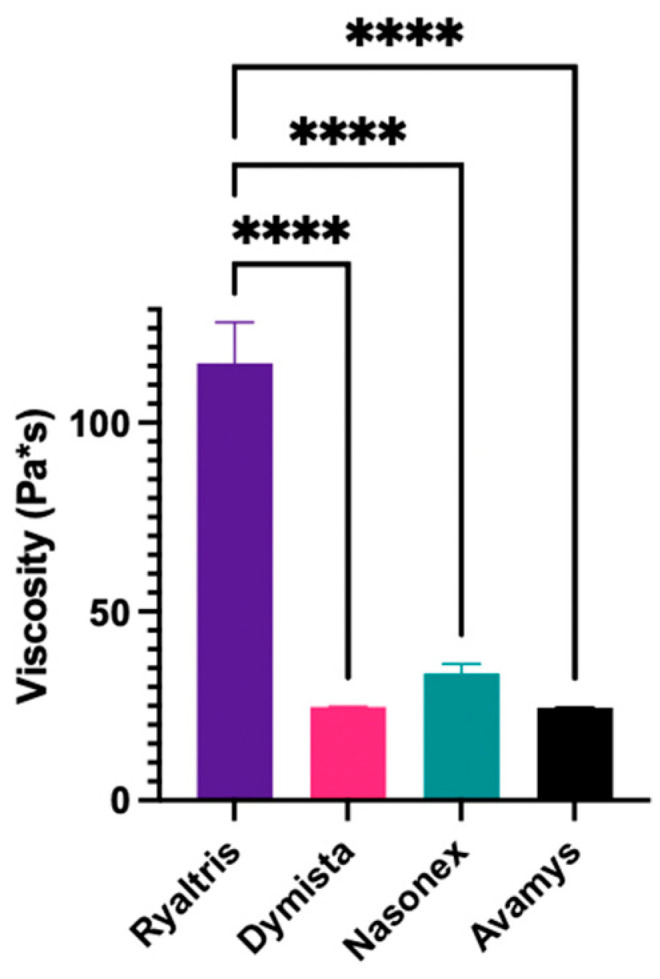
Average viscosity of the formulations contained in the four commercial products under investigation. **** *p* < 0.0001.

The average viscosity values obtained by analyzing the liquid formulations of the four commercial nasal products under investigation are shown in Figure 1.

The ANOVA test showed the viscosity measurements to be highly statistically different (*p* value = 0.0001). In particular, Ryaltris showed the highest viscosity compared to the other products which presented very low viscosity.

#### 3.1.2. Formulation Flow on Inclined Plane

**Figure 2:**
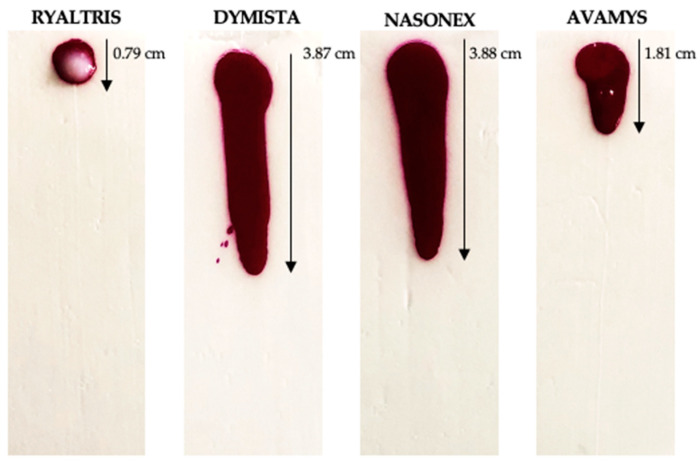
Flow on an inclined plane of all tested formulations in contact with a semisolid substrate.

In order to complement the viscosity test, a simple experiment to evaluate the flow of the formulation on an inclined plane could provide further information about the tendency of the nasal product to drip off after the administration. In order to carry out the flow/adhesion studies, images were taken one minute after deposing the product on an inclined plane coated with a semisolid gel, in this case, a water-finding paste that despite being water-free contains hydrophilic components and changes color in contact with water, facilitating the measurement of the formulation movement. The flow patterns created by Ryaltris, Dymista, Nasonex, and Avamys are shown in Figure 2.

Interestingly, results only partially were determined by the formulation viscosities. Dymista was shown to provide the faster flow, as it was shown to be not very viscous. At the same time, Ryaltris, the most viscous suspension, did not show any appreciable flow, but remained attached to the semisolid coating-placed inclined plane. Avamys showed an intermediate behavior with a moderate flow. Contrary to expectation, Nasonex provided a flow pattern similar to Dymista, somewhat in contrast to the different viscosity values measured with the two products.

### 3.2. Device-Related Aspects

#### 3.2.1. Spray Droplet Size Distribution

All the commercial products, Ryaltris, Dymista, Nasonex, and Avamys, were subjected to spray droplet size analysis.

**Figure 3:**
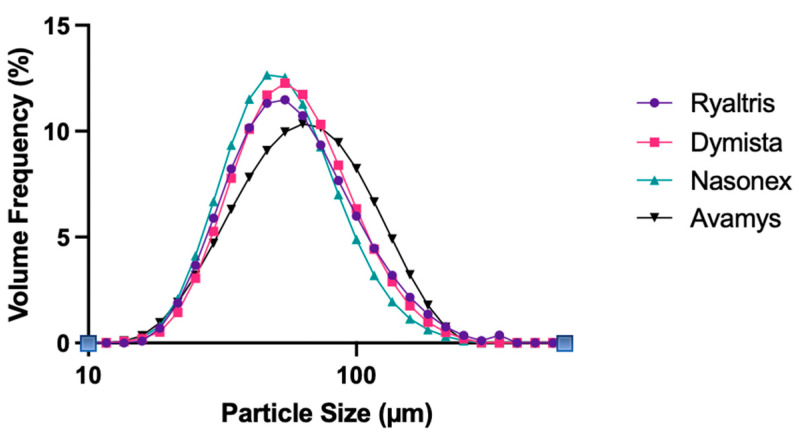
Droplet size distribution of nasal sprays expressed by volume.

The average droplet size distributions of all the nasal products are presented in Figure 3, while Table 3 shows the Dv10, Dv50, and Dv90 diameters, corresponding to droplet size relating to the 10%, 50%, and 90% percentile of droplet population on a volume basis, and SPAN, a value expressing distribution width.

The droplet size distributions were found to be very similar with a peak around 50–60 µm, with Avamys presenting overall the largest droplets. In any case, the Dv10, Dv50, and Dv90 values were not significantly different among all the nasal sprays tested. Nasonex was the one with the narrower droplet size distribution (lowest SPAN), while Avamys and Ryaltris presented the wider spray distribution (highest SPAN values), but again, those differences were not statistically significant.

#### 3.2.2. Nasal Cast Deposition

The analysis of nasal cast deposition was carried out on all four formulations. The deposition patterns of Ryaltris, Dymista, Nasonex, and Avamys were investigated with the administration of the product in a silicone nasal cast coated with a water-finding paste. The device was inserted in the cast nostril at a 45° angle from the horizontal to a patient’s “normal” handling of the device.

For each product, the fraction of nasal surface covered by the spray was expressed as a percentage of the total, as well as the fraction of spray deposited in each of the three regions of interest (ROI) considered, i.e., nasal vestibule, upper and middle turbinate, and lower turbinate (see Figure S2 in the Supplementary Materials).

**Figure 4:**
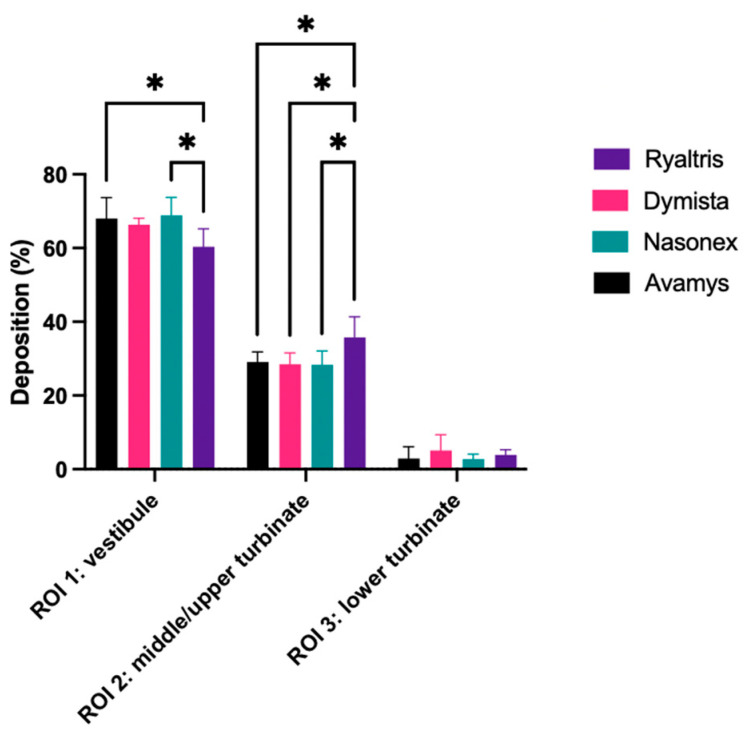
Regional deposition percentiles in nasal vestibule (ROI 1), middle–upper turbinate (ROI 2), and lower turbinate (ROI 3) for the five nasal products tested using a 45° actuation angle. * *p* < 0.05.

The total deposition of Avamys was 27.42 ± 1.72%, 28.96 ± 2.86% for Dymista, 27.74 ± 1.59% for Nasonex, and 28.69 ± 2.41% for Ryaltris. Even if the total deposition area of the products was found to be slightly different, overall results were not statistically significant (*p* = 0.28). Interestingly, the analysis of regional deposition resulted in some slight but statistically relevant differences, denoting for Ryaltris a lower deposition in the anterior region of the nasal vestibule and a larger deposition in the upper and middle turbinate region (Figure 4).

**Figure 5:**
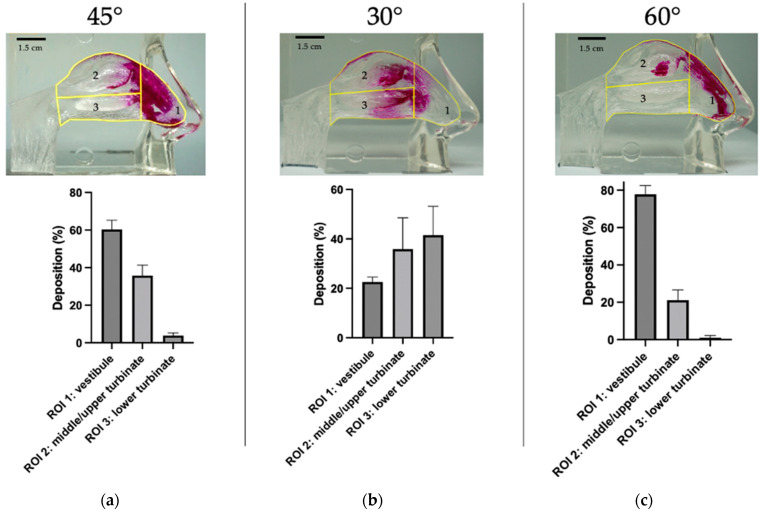
Deposition patterns (upper images) and analysis of regional deposition percentages (lower graphs) of Ryaltris when activated with a (**a**) 45°, (**b**) 30°, and (**c**) 60° angle from the horizontal (*n* = 3 ± SD).

Ryaltris nasal spray was also analyzed to investigate potential differences in the deposition pattern and regional deposition in the nasal cast when changing the inclination of the product during actuation from 45° to 30° or 60° angles from the horizontal plane, hence simulating an incorrect use of the device. The deposition patterns of the formulation that were subjected to the test with three different actuation angles are pictured in Figure 5.

The total nasal area covered by the spray when it was activated with a 30° angle was of 28.23 ± 5.30% and the nasal area covered by the spray when activating the device with a 60° angle was of 18.34 ± 0.88%. Both were compared to the nasal area covered when correctly handling the Ryaltris device of 28.69 ± 2.41%. The deposition analysis in the nasal cast demonstrated that a 60° angle actuation led to a significantly higher deposition in the nasal vestibule (*p* = 0.0049) and a significantly lower deposition in the middle–upper turbinate (*p* = 0.0190). Despite that, there was not a significant difference in the total area covered with 45° and 30° angle tests, as different distribution in the regions of interest was observed with a wider coverage in terms of deposition for the 30° angle, with significantly lower anterior deposition in the vestibule (*p* < 0.0001) and significantly higher deposition in the lower turbinate (*p* < 0.0001).

### 3.3. Active Ingredient-Related Aspects

#### 3.3.1. Dissolution Test Using Respicell Apparatus

**Figure 6:**
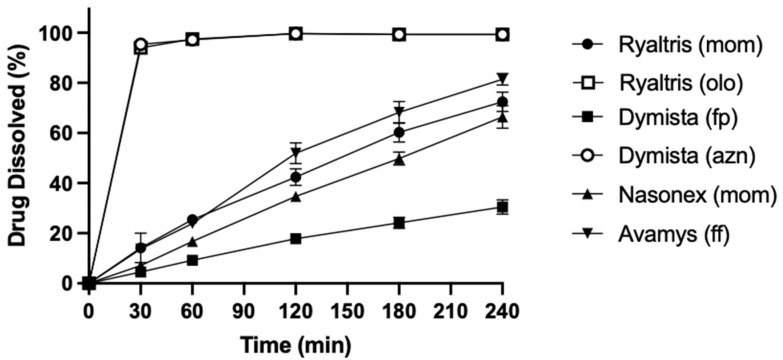
Dissolution profiles of the active ingredients of the nasal products tested (ff = fluticasone fumarate; fp = fluticasone propionate; mom = mometasone propionate; azn = azelastine HCl; olo = olopatadine HCl).

The dissolution studies of the nasal products were performed using Respicell, an apparatus designed for the dissolution of inhalation products that allows the product wetting and dissolution process to happen at the wet surface of a polymeric membrane used to separate the donor and acceptor compartment. The dissolution profiles are outlined in Figure 6. The figure also presents as controls the profiles of the two soluble active ingredients present in Dymista and Ryaltris, i.e., azelastine and olopatadine, demonstrating that the membrane separating the compartments (0.4 µm pore size polycarbonate membrane) does not restrict the diffusion of molecules once solubilized. The dissolution profiles for the corticosteroid showed a slightly faster dissolution profile for fluticasone furoate present in Avamys, followed by mometasone furoate of Ryaltris and Nasonex, while Dymista fluticasone propionate showed a slower dissolution with less than 30% of the dose dissolved within 240 min.

Dissolution profiles were compared using the difference (*f*_1_) and similarity (*f*_2_) factors, a statistical approach well established for the comparison of dissolution profiles of oral products, and for each pair of products, *f*_1_ and *f*_2_ were calculated (Table 4). From the calculation of *f*_1_ and *f*_2_ for each comparison, it was possible to highlight that Avamys, Nasonex, and Ryaltris had a similar dissolution profile, as *f*_1_ values were below 15 and *f*_2_ values above 50. On the contrary, the slower dissolution profile evidenced with Dymista was confirmed to be different (*f*_1_ > 15) and dissimilar (*f*_2_ > 50) from the other three products.

#### 3.3.2. Suspended Particle Size Distribution and Morphology Analysis

**Figure 7:**
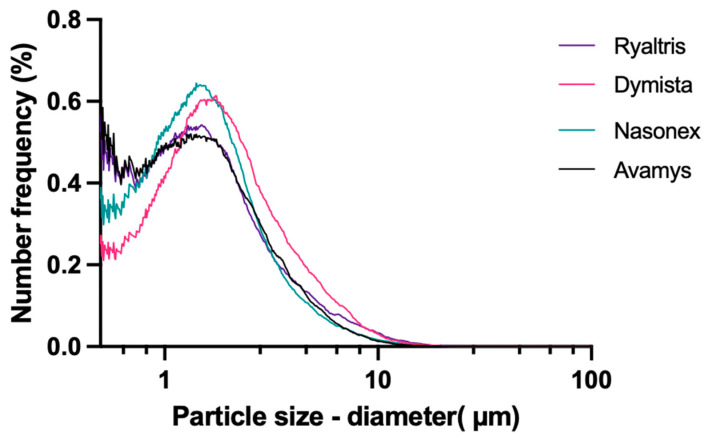
Number-based particle size distributions of suspended particles in the nasal products (1:10 diluted samples, average of 3 measurements).

The nasal products containing corticosteroids are suspensions; in addition, all products contained cellulose that is also present as dispersed insoluble particles. Figure 7 presents the suspended particle size distribution by number, while Table 5 shows the 10, 50, and 90% percentile of the particle number distribution (Dn10, Dn50, and Dn90).

The suspended particle distribution presented for all products a main peak between 1 and 2 µm, while it differed for the frequency by a number of particles smaller than 1 µm. Ryaltris and Avamys presented the highest numbers of submicron particles, followed by Nasonex, while Dymista was the product with the smallest presence of extra small particles.

These differences in particle size distribution are reflected by the circular equivalent diameter values presented in Table 5. In particular for Dn10, Ryaltris and Avamys show a smaller, even if not statistically significant, particle size than Nasonex and Dymista. Also, in the case of Dn50, Dymista appears to have the highest particle size, even if again the difference is not statistically significant.

**Figure 8:**
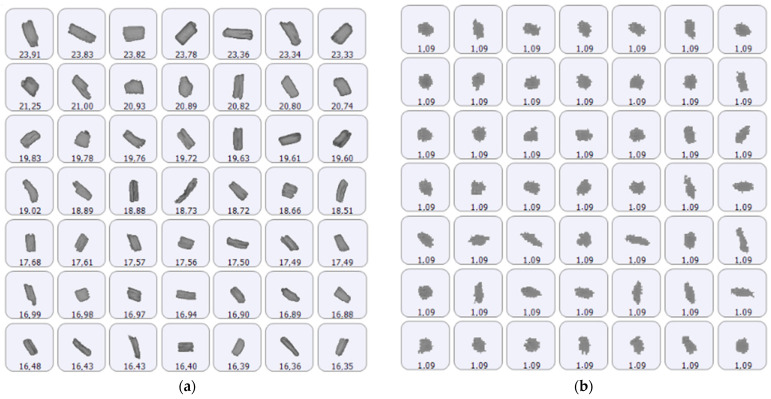
Representative sample of the suspended particles imaged for Ryaltris: (**a**) largest particles and (**b**) particles around the main peak of the number distribution. The number under each particle indicates the projected area circular equivalent diameter in micrometers.

It is interesting to notice that the Morphologi instrument also provides images of the particles that are measured and this morphological analysis allows us to comment more on the nature of the particles measured. In Figure 8, two samples of Ryaltris particle images are presented as an example since similar data were observed for the other product (see Supplementary Materials, Figures S9–S11).

In fact, it could be evidenced a difference in morphology between larger and smaller particles. Among large particles of the population distribution (Figure 8a), a fibrous, elongated morphology, attributable to cellulose particles, is more common. On the contrary, among smaller particles (Figure 8b), fewer particles appear elongated, while irregular but more circular particles are prevalent, potentially indicating a larger presence of the micronized active ingredient in the smaller fraction of the particle population.

## 4. Discussion

Among different physico-chemical characteristics of liquid nasal formulations, viscosity is invariably indicated as one of those parameters that are pivotal for the performance of the nasal products affecting droplet particle size, spray angles, and deposition as well as the residence time of the product after deposition [2,21]. The nasal products tested present different formulation compositions (see Table 4) and, as a consequence, show significantly different viscosities. Taking the classic nasal spray Nasonex as a reference, it is noticeable that the viscosity of the combination preparation Dymista is only a small fraction of its viscosity, while the viscosity of Ryaltris is 4–5 times higher and thus tens of times higher than the viscosity of the other combination preparations.

However, these aforementioned differences produced a remarkably insignificant effect on the droplet size distribution of the sprays analyzed, with three products having almost superimposable droplet size distribution, while only Avamys presented a slightly higher particle size. Indeed, there are no significant differences in the droplet size of the four nasal sprays and the lower limit of the droplet size, as characterized by the Dv10, differs only marginally between the four preparations. The absolute value of just under 30 µm ensures that the preparations cannot penetrate the lower respiratory tract even in the event of accidental inhalation.

The lack of impact of viscosity on droplet size distribution is in contrast with the general conclusions of the work of Trows et al. that using a range of formulations containing 1–5% sodium carboxymethyl cellulose and 0.0001–0.1% polysorbate 80, confirmed that viscosity enhancement has a major influence on droplet size distribution determining an increase in particle size, while surface tension has almost no effect [22]. However, in most studies looking at the impact of formulation characteristics, viscosity as well as other parameters are varied keeping constant the device, while here the matching between different formulations and different devices could have led to similar spray performances.

The high similarity in the droplet size distributions resulted in an almost identical deposition in the nasal cast for the four nasal products both in terms of area and pattern, with a slight but significant difference in the regions of deposition evidenced only for Ryaltris, that favored a deeper penetration of the spray to the middle–upper turbinate. The deposition in the nasal cavity at an application angle of 45° shown in Figure 4 confirms this by showing that the deposition of Ryaltris on the middle and upper nasal concha is more relevant than with the three other nasal sprays tested. This capacity of deposing the droplets deeper in the nasal cavity was confirmed by a test performed at a 30° actuation angle instead of 45°. Here, an optimal distribution in the nasal cavity is shown at the spray angle of approx. 30°, which is also recommended in the guidelines. However, even when the angle is increased to 45°, the essential parts of the nasal cavity, namely the middle and upper turbinates, are exposed more strongly. This should be clearly communicated to the patient by the prescribing doctor or recommending pharmacist.

Interestingly, Foo and co-workers in their study tried to elucidate the impact of formulation, device, and actuation parameter interaction on the deposition in an MRI-derived nasal cavity replica and found that viscosity, droplet size, and device had minimal effects. On the contrary, the actuation angle together with the emitted spray plume angle were the factors determining the deposition efficiency [23].

More in line with the expectations, the viscosity had a major impact on the flow of the formulation on an inclined plane with results clearly differentiating the nasal products (Ryaltris < Avamys < Dymista~Nasonex). This is of great clinical implication since, as Figure 2 shows, the novel combination spray, i.e., Ryaltris, remains at the site of deposition and does not flow due to the choice of a higher viscosity. This enables an intensive exchange with the nasal mucosa. With the other combination preparation, Dymista, it can be assumed that a considerable proportion of the spray volume either drips out of the nose through the vestibule or flows into the pharynx via the choanae. In this test, the only result that was not following the formulation viscosities measured was the similar dripping evidenced for Dymista and Nasonex. However, these products were the only two containing glycerol, an aspect that could have influenced the interaction with the semisolid substrate coating the inclined plane, i.e., a water-free gel paste containing polyethylene oxide and propylene glycol as main components. This test is generally indicated as a potential indication of the formulation to drip off the nose or to flow rapidly toward the throat; the relevance of the test is strongly related to the type of mucus simulant used [12]. In a work in which two different coatings were used, one simulating healthy runny mucus, containing porcine mucin, and another mimicking thick diseased mucus, containing locust bean gum and sodium dodecyl sulfate, it was shown that, in general, increasing the concentration of microcystalline cellulose in the formulation tested, a reduction of the dripping was observed; however, for the diseased mucus there was a different behavior for almost all formulations tested with a faster dripping speed compared to healthy mucus or no coating, and this was attributed to a different interaction with the mucus simulant [24].

As in the case of inhalation products, the methods and apparatuses for dissolution testing of nasal products are not standardized and described in pharmacopoeial monographs. Moreover, this is generally a test considered for nasal powders, gels, or nanocarriers, despite also in the case of suspensions the dissolution is a fundamental step for the bioavailability of the active ingredient. In most of the studies, compendial methods originally described for oral dosage forms are adapted to test nasal products [25]. This has, however, the fundamental problem that the thickness of the mucus layer in the nasal cavity is just 15 µm and the volume of liquid available for dissolution is limited [26]. For this reason, the Respicell apparatus was used for the first time in this study to perform the dissolution of the poorly soluble corticosteroids present in the four commercial nasal products selected. This innovative apparatus, which has already been demonstrated to be highly discriminating in dissolution testing of inhalation products, simulates the limiting conditions present at the mucosal surface, obliging the formulation to dissolve at the liquid surface separated from the bulk dissolution medium by a polymeric membrane [19].

The results show that both antihistamines, olopatadine and azelastine, already in solution in the products, appear very quickly (within a few minutes) in the acceptor compartment and therefore are likely to translate in a rapid onset of action at the target site, i.e., the nasal mucosa. The situation is somewhat different for ingredients from the corticosteroid group. The results showed that one product, i.e., fluticasone propionate in Dymista, dissolved more slowly compared to the other corticosteroids, i.e., mometasone propionate in Ryaltris and Nasonex and fluticasone furoate in Avamys. This could be partially attributed to some differences in the suspended particle size distribution, that showed for Dymista the lowest fraction of particles below 1 µm, while that fraction was the highest in the case of Avamys and Ryaltris that showed the fastest dissolution rates. But it is undeniable that the physico-chemical properties of the active ingredient also have a critical role in the dissolution kinetics, since the two products containing mometasone furoate, i.e., Ryaltris and Nasonex showed the most similar dissolution profiles. Interestingly, it has been reported that the onset of symptom relief is approximately 7 h of mometasone furoate [27] and 8 h for fluticasone furoate [28,29] in patients with allergic rhinitis. On the contrary, in a comparative study between nasal sprays in the treatment of Japanese cedar pollinosis, the ones containing fluticasone furoate showed onset of action at day 1, while those containing fluticasone propionate showed efficacy from the second day of treatment [30]. Variations in effect according to onset time may be attributed to differences in the study set-up concerning the record of pharmacological activity but also to the underlying pathology, which in turn influence the type and composition of epithelial airways secretions [31]. Consequently, these alterations can modify the biological interface properties where the active ingredient has to dissolve, potentially leading to a delayed effect. Therefore, investigating the behavior of active principles in the presence of mucus models mimicking the physiological environment is crucial [32].

Very recently, the group of Günther Hochhaus at the University of Florida has supported the hypothesis that suspension nasal formulations differing in the API particle size differ in their bioavailability. Two mometasone furoate formulations with different particle size and presenting different dissolution profiles in vitro were clearly not bioequivalent with regard to pharmacokinetics parameters such as AUC and C_max_ [33]. These results open the way to new approaches in the development and characterization of nasal products, with an emphasis on tests more focused on the understanding of the behavior of the product at the biological interface, necessary to complete the traditional test focusing on the technical aspects of the product performance.

In vitro tests for the characterization of nasal products surely have some limitations. Indeed, it is complex to replicate the environment of the human nasal cavity including physiological and biochemical conditions. Furthermore, the interaction of nasal formulations with the nasal mucosa or toxicity effects is challenging to assess accurately in vitro. These limitations, indeed, highlight the need to combine in vivo to predict the behavior of the drugs and to in vivo assays testing effectiveness and toxicity. Therefore, possible clinical implications should be further validated by the means of randomized controlled clinical trials.

## 5. Conclusions

The characterization of anti-allergic nasal sprays required by the pharmacopoeias and regulatory agencies’ guidance represents a necessary but incomplete approach to the complexity of such products. In the present work, the application of a combination of classic and advanced methods clearly demonstrates that a number of relevant properties related to nasal spray deposition and drug dissolution at the region of interest for action or absorption, i.e., the nasal mucosa, could be obtained (see Supplementary Materials, Figure S12).

The results of the experimental studies on the four nasal sprays obtained here are expected to have important clinical implications, both with regard to the application of the sprays by the patient and with regard to their interaction with the mucosa of the nasal cavity, i.e., their distribution in the nasal cavity and their persistence on the mucosa.

Ryaltris nasal spray, a fixed dose combination of mometasone furoate and olopatadine hydrochloride, showed peculiar properties when compared to the other commercial products analyzed (Avamys, Nasonex, Dymista). Ryaltris had a higher viscosity, resulting in an almost absent dripping in an inclined plane test. This feature may result in a longer retention time in the nasal cavity without affecting the nasal deposition. On the contrary, Ryaltris nasal deposition was demonstrated to be deep and uniformly distributed on turbinates, especially at a 30° angle of insertion of the device in a nasal cast. Finally, the dissolution profiles showed that the mometasone furoate contained in the Ryaltris combination spray dissolved more rapidly than the fluticasone propionate in the Dymista spray by a factor of two and similarly to other comparators, i.e., mometasone furoate and fluticasone furoate contained in Nasonex and Avamys, respectively. This could have possible clinical implications resulting in a faster onset of action and efficacy of the novel combination preparation Ryaltris, considering also the higher viscosity of the formulation.

These aspects appear relevant not only for the characterization and differentiation of nasal products on the market, as demonstrated here, but even more for the development of novel nasal products, offering a better understanding of their clinical performance [10,13,34].

## Figures and Tables

**Table 1:** Composition of nasal spray products under investigation.

Nasal Product (Batch)	Active Substance	Dose per Actuation (µg)	Excipients
Ryaltris (12220061A)	Mometasone furoate Olopatadine HCl	25 665	Microcrystalline cellulose Na_2_HPO_4_·7H_2_O Na carboxymethyl cellulose Polysorbate 80 Benzalkonium chloride NaCl, EDTA, Water for injections
Dymista (IC20087)	Fluticasone propionate Azelastine HCl	50 137	Glycerol Microcrystalline cellulose Na carboxymethyl cellulose Polysorbate 80 Benzalkonium chloride Phenylethyl alcohol EDTA, Purified water
Nasonex (UO13904)	Mometasone furoate	50	Dispersible cellulose Glycerol Sodium citrate, Citric acid Polysorbate 80 Benzalkonium chloride Purified water
Avamys (P87R; 7U8Y)	Fluticasone furoate	27.5	Anhydrous glucose Dispersible cellulose Polysorbate 80 Benzalkonium chloride EDTA, Purified water

**Table 2:** Details of HPLC methods for the APIs contained in the nasal products under investigation.

Active Substance (API)	Column	Wavelength (nm)	Temperature (°C)	Mobile Phase	Injection Volume (µL)	Flow Rate (mL/min)
Fluticasone furoate	Zorbax Eclipse XD8-C18 5 µm 4.6 × 150 mm (Agilent, Santa Clara, CA, USA)	238	40	Gradient ^1^ 0.02 M NaH_2_PO_4_ Phosphate buffer (pH 3)/Acetonitrile	50	1.0
Fluticasone propionate Azelastine HCl		238 210	40	Gradient ^1^ 0.02 M NaH_2_PO_4_ Phosphate buffer (pH 3)/Acetonitrile	50	1.0
Mometasone furoate Olopatadine HCl	Zorbax Eclipse XDB-C8 5 µm 4.6 × 150 mm (Agilent, Santa Clara, CA, USA)	248	25	Isocratic Methanol/Water 60:40	100	1.2
Mometasone furoate		248	25	Isocratic Methanol/Water 60:40	100	1.2

^1^ 0.02 M Phosphate buffer/ACN = 75:25 (0–4 min), 25:75 (5–10 min), 75:25 (11–12 min).

**Table 3:** Average volume diameters and SPAN of the nasal spray droplet size distributions.

Products	Dv10 (µm)	Dv50 (µm)	Dv90 (µm)	SPAN
Ryaltris	27.82 ± 1.06	53.23 ± 4.35	112.70 ± 21.25	1.57 ± 0.30
Dymista	28.78 ± 1.61	52.78 ± 5.77	99.55 ± 5.77	1.31 ± 0.30
Nasonex	26.91 ± 1.53	49.15 ± 5.34	91.77 ± 21.25	1.29 ± 0.30
Avamys	28.28 ± 2.91	58.62 ± 3.98	119.03 ± 5.09	1.55 ± 0.11

**Table 4:** Difference (*f*_1_) and similarity (*f*_2_) factors calculated for dissolution profiles.

Nasal Products	Difference Factor (*f*_1_)	Similarity Factor (*f*_2_)
Ryaltris vs. Avamys	4.41	83.11
Dymista vs. Ryaltris	58.68	30.30
Dymista vs. Nasonex	98.19	44.35
Nasonex vs. Ryaltris	13.84	61.34
Avamys vs Dymista	97.56	31.55
Avamys vs. Nasonex	11.40	64.50

**Table 5:** Average number diameters of the nasal product suspended particle size distributions.

Products	Dn10 (µm)	Dn50 (µm)	Dn90 (µm)
Ryaltris	0.56 ± 0.02	1.33 ± 0.05	3.78 ± 0.35
Dymista	0.68 ± 0.02	1.65 ± 0.24	4.14 ± 0.85
Nasonex	0.61 ± 0.01	1.39 ± 0.14	3.22 ± 0.52
Avamys	0.56 ± 0.06	1.27 ± 0.19	3.38 ± 0.37

## Data Availability

The datasets presented in this article are not readily available because they are the property of A. Menarini Industrie Farmaceutiche Riunite SrL (a company of the Menarini Group). Requests to access the datasets should be directed to S.B.

## Supplementary Materials

The following supporting information can be downloaded at: https://www.mdpi.com/article/10.3390/pharmaceutics16080989/s1, Table S1: Physico-chemical properties of active ingredients; Figure S1: Representation of the products under study; Figure S2: Deposition in the nasal cast; Figure S3: Calibration curve mometasone furoate; Figure S4: Calibration curve fluticasone propionate; Figure S5: Calibration curve fluticasone furoate; Figure S6: Calibration curve of olopatidine hydrochloride; Figure S7: Calibration curve of azelastine hydrochloride; Figure S8: Images of a representative sample of the suspended particles of Avamys; Figure S9: Image of Respicell apparatus; Figure S10: Images of a representative sample of the suspended particles of Dymista; Figure S11: Images of a representative sample of the suspended particles of Nasonex; Figure S12: Representation of advanced test for nasal spray characterization.
