# Supplementary material for: Clinically Relevant Characterization and Comparison of Ryaltris and Other Anti-Allergic Nasal Sprays
Source: Pharmaceutics. 2024 Jul 26;16(8):989. doi: 10.3390/pharmaceutics16080989 (PMC11357686; doi:10.3390/pharmaceutics16080989)
Supplement: Supplementary file 1 [file pharmaceutics-16-00989-s001.zip › pharmaceutics-3077683-supplementary.pdf]

Supplementary material
"**Advanced Characterizations and Dissolution Test for Decongestant Nasal Products**"
Patterlini *et al.* Pharmaceuticals, 2024

Physicochemical properties of the products under studying

The analysed products are presented in **Figure S1,** and the physicochemical properties (molecular weight, melting point, and octanol/water partition coefficient) of the active ingredients are listed in Table S1.

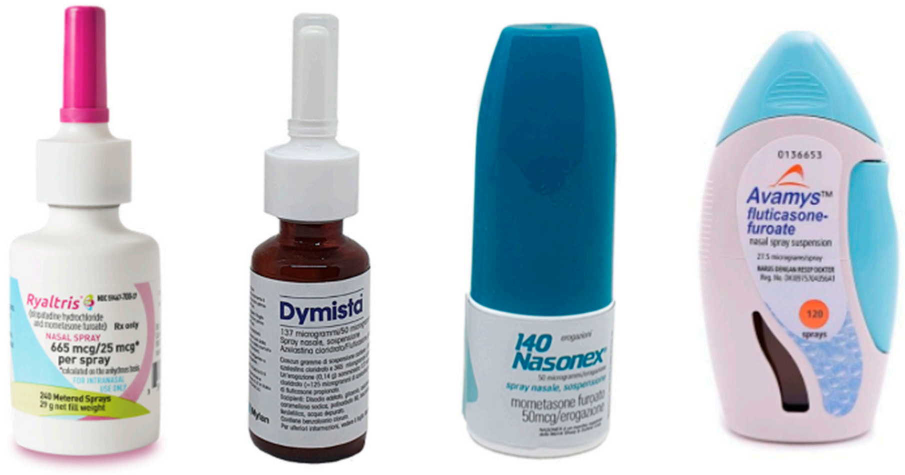

Figure S1: *Representation of the products under study*

Table S1*: physicochemical properties of active ingredients*

	**Molecular weight (g/mol)**	**Melting point**	**Octanol/water partition coefficient (logP)**
Mometasone Furoate	521.4	215-228	4.115
**Fluticasone Propionate**	444.5	272- 273	2.78
**Fluticasone Furoate**	538.6	250-252	4.13

Nasal cast deposition

Regions of interest in the nasal cast deposition experiments carried out on nasal products (Figure S2).

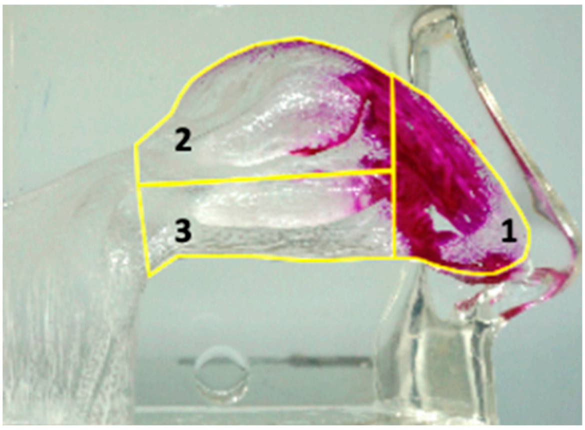

Figure S2: *deposition in the nasal cast was analysed dividing the cavity in three regions of interest: 1. Nasal vestibule; 2. Middle and upper turbinates; 3. Lower turbinate.*

Validation Parameters of the HPLC methods

The quantification of the mometasone furoate into the samples (Nasonex and Ryaltris) was performed using the HPLC methods reported in manuscript's Table 2.

A stock solution (50 µg/mL) was prepared by weighting 5 mg of mometasone furoate powder in 100 ml of methanol into a glass flask.

Starting from the stock solution a calibration curve was built in the range of 2 – 50 µg/ml. The samples were diluted using methanol as solvent.

The LOD and LOQ calculated from triplicate of the calibration curve obtained were respectively 0.06 µg/ml and 0.19 µg/ml.

The calibration curve achieved for mometasone furoate is presented in Figure S3.

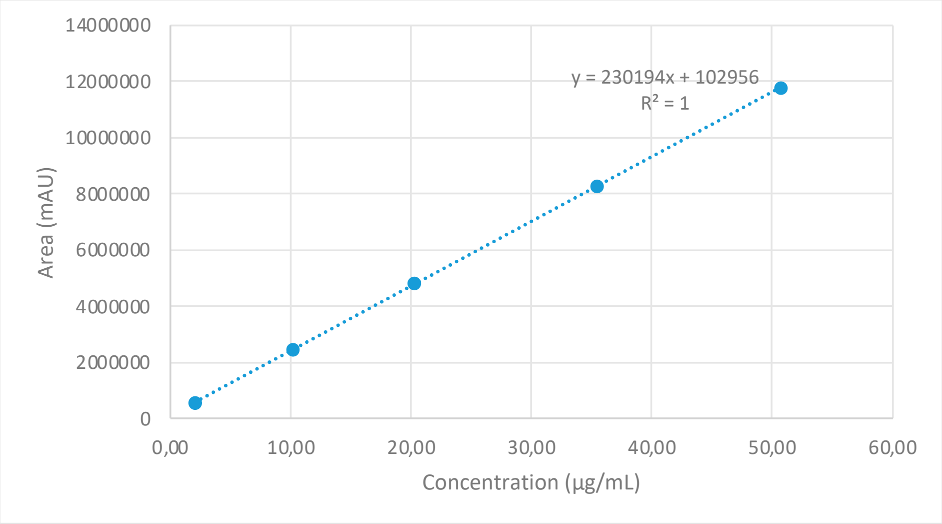

Figure S3*: calibration curve of mometasone furoate.*

The quantification of the fluticasone propionate in samples (Dymista) was performed using the HPLC method reported in manuscript's Table 2.

A stock solution (50 µg/mL) was prepared by weighting 5 mg of fluticasone propionate powder in 100 ml of ACN:H_2O (60:40) into a glass flask.

Starting from the stock solution a calibration curve was built in the range of 5– 49 µg/ml. The samples were diluted using ACN:H_2O (60:40) as solvent.

The LOD and LOQ calculated from triplicate of the calibration curve were respectively 0.32 µg/ml and 0.96 µg/ml.

The calibration curve achieved for fluticasone propionate is presented in Figure S4.

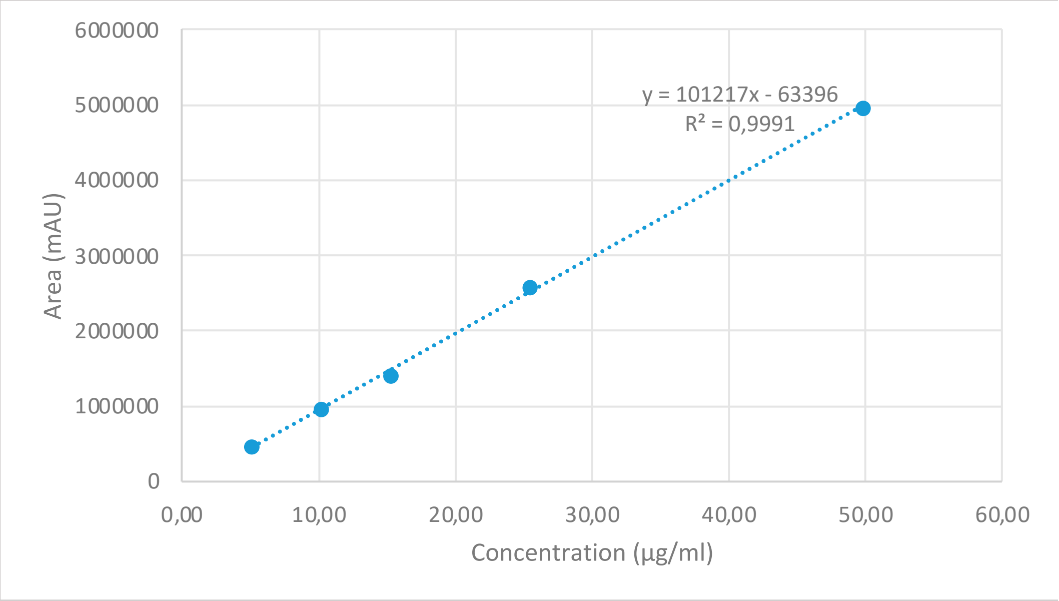

***Figure S4**: calibration curve of fluticasone propionate.*

The quantification of the fluticasone furoate in samples (Avamys) was performed using the HPLC method reported in manuscript's Table 2. A stock solution (50 µg/mL) was prepared by weighting 5 mg of fluticasone furoate powder in 100 ml of ACN:H_2O (60:40) into a glass flask. Starting from the stock solution a calibration curve was built in the range of 2– 50 µg/ml. The samples were diluted using ACN:H_2O (60:40) as solvent.

The LOD and LOQ calculated from triplicate of the calibration curve were respectively 2.38 µg/ml and 7.22 µg/ml.

The calibration curve achieved for fluticasone furoate is presented in Figure S5.

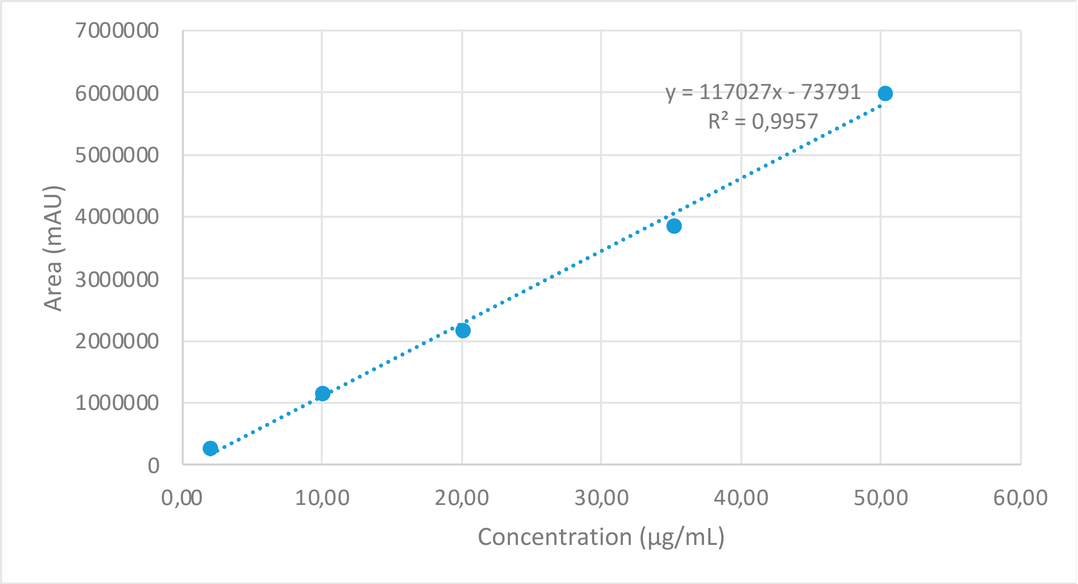

***Figure S5**: calibration curve of fluticasone furoate.*

The quantification of the Olopatidine hydrochloride in samples (Ryaltris) was performed using the HPLC method reported in manuscript's Table 2.
A stock solution (200 µg/mL) was prepared by weighting 5 mg of fluticasone propionate powder in 25 ml of methanol into a glass flask.
Starting from the stock solution a calibration curve was built in the range of 2– 192 µg/ml. The samples were diluted using methanol as solvent.
The LOD and LOQ calculated from triplicate of the calibration curve were respectively 0.44 µg/ml and 1.34 µg/ml.
The calibration curve achieved for fluticasone propionate is presented in Figure S6.

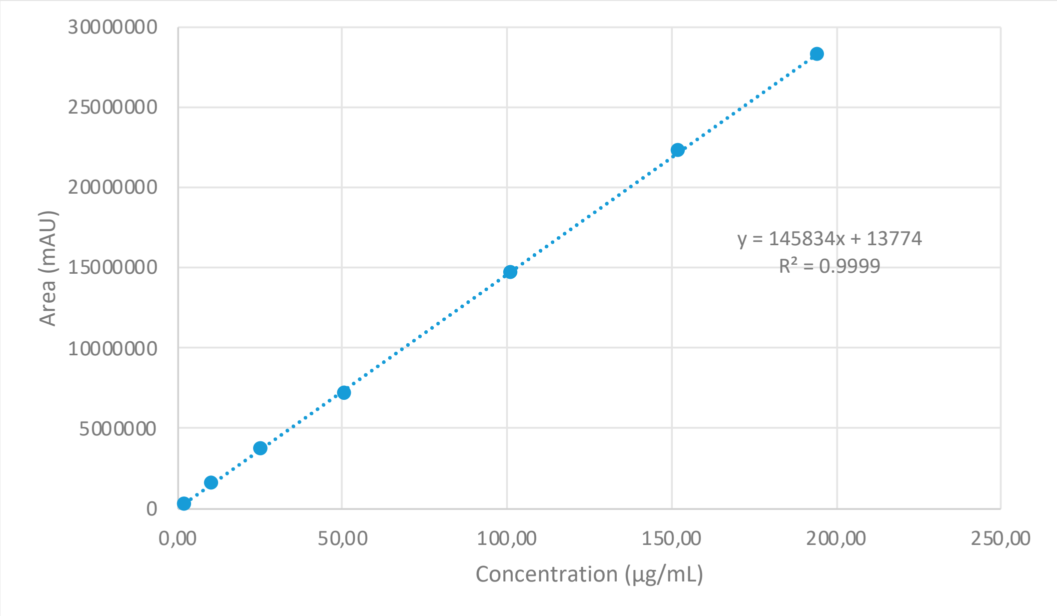

***Figure S6**: calibration curve of olopatidine hydrochloride.*

The quantification of the azelastine hydrochloride in samples (Dymista) was performed using the HPLC method reported in manuscript's Table 2.
A stock solution (100 µg/mL) was prepared by weighting 5 mg of fluticasone propionate powder in 10 ml of ACN:H_2O (60:40) into a glass flask. A work solution (100µg/ml) was prepared by diluting the stock solution.
Starting from a work solution (100µg/ml) a calibration curve was built in the range of 2–98 µg/ml. The samples were diluted using ACN:H_2O (60:40) as solvent.
The LOD and LOQ calculated from triplicate of the calibration curve were respectively 0.34 µg/ml and 1.02 µg/ml.
The calibration curve achieved for fluticasone propionate is presented in Figure S7.

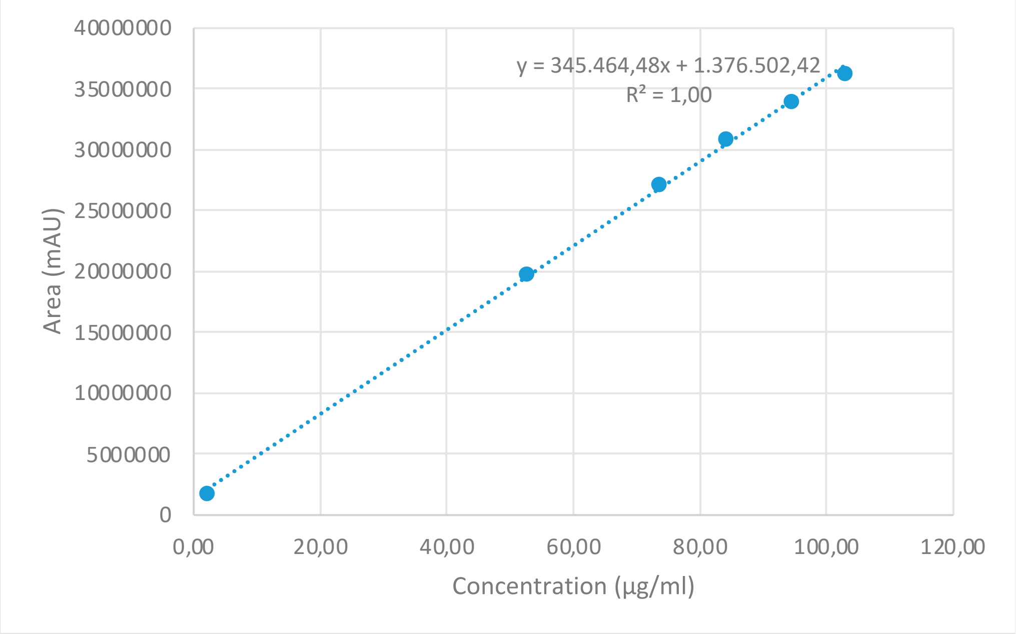

***Figure S7**: calibration curve of azelastine hydrochloride.*

Dissolution test

Respicell® apparatus for vertical dissolution is presented in Figure S8.

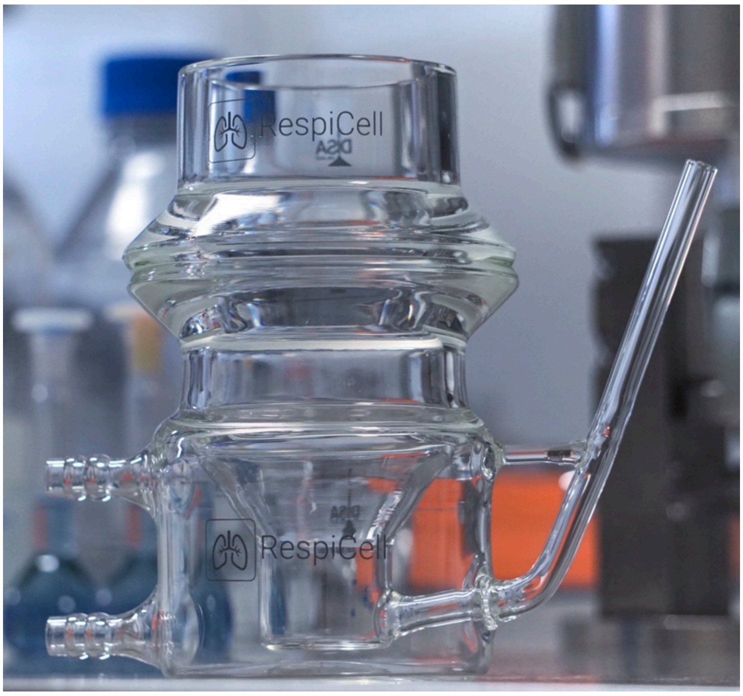

***Figure S8:** Respicell apparatus for vertical dissolution test.*

Morphologi analysis

Images of the particles of all the products measured by morphologi are presented in the followed Figures (Figure S9-S11).

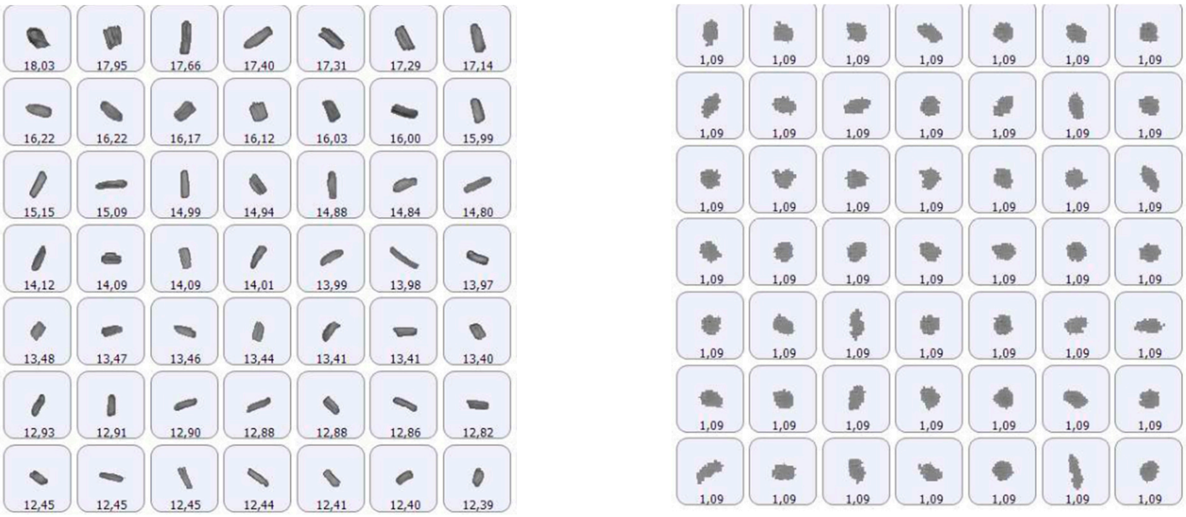

(a) *(b)*

Figure S9*: representative sample of the suspended particles imaged for Avamys: (a) largest particles and (b) particles around the main peak of the number distribution. The number under each particle indicate the projected area circular equivalent diameter in micrometres.*

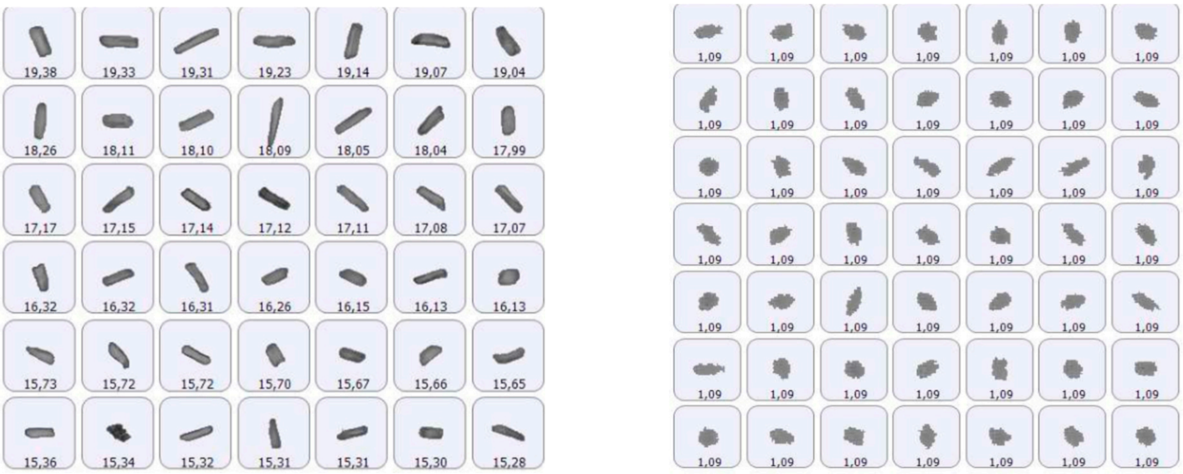

(a) (b)

Figure S10*: representative sample of the suspended particles imaged for Dymista: (a) largest particles and (b) particles around the main peak of the number distribution. The number under each particle indicate the projected area circular equivalent diameter in micrometres.*

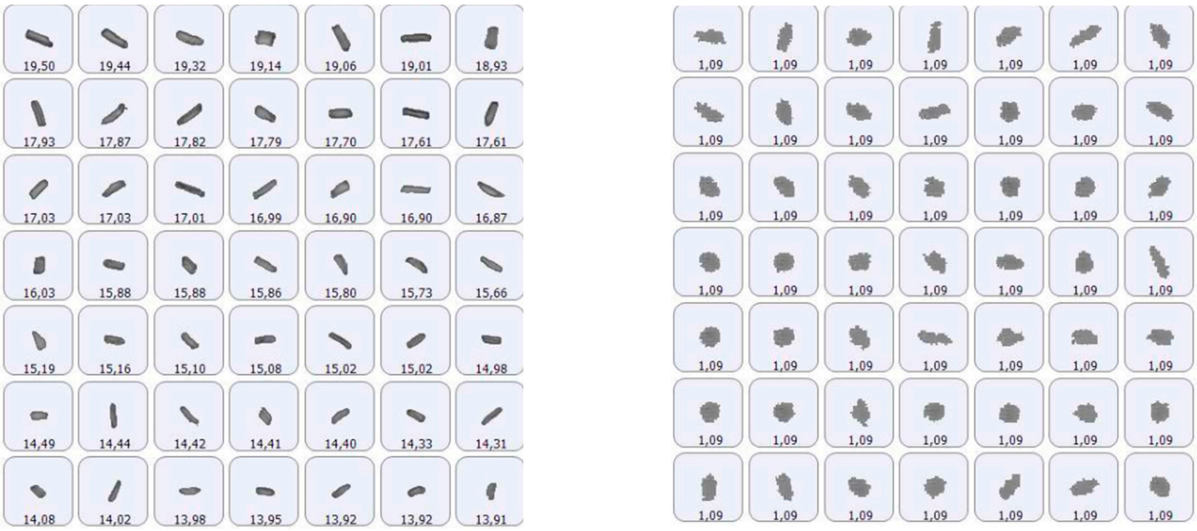

(a) (b)

***Figure S11**: representative sample of the suspended particles imaged for Nasonex: (a) largest particles and (b) particles around the main peak of the number distribution. The number under each particle indicate the projected area circular equivalent diameter in micrometres.*

Advanced tests for a thorough assessment

In Figure S12 are presented the advanced preclinical tests that should be used for thorough assessment of corticosteroid nasal sprays.

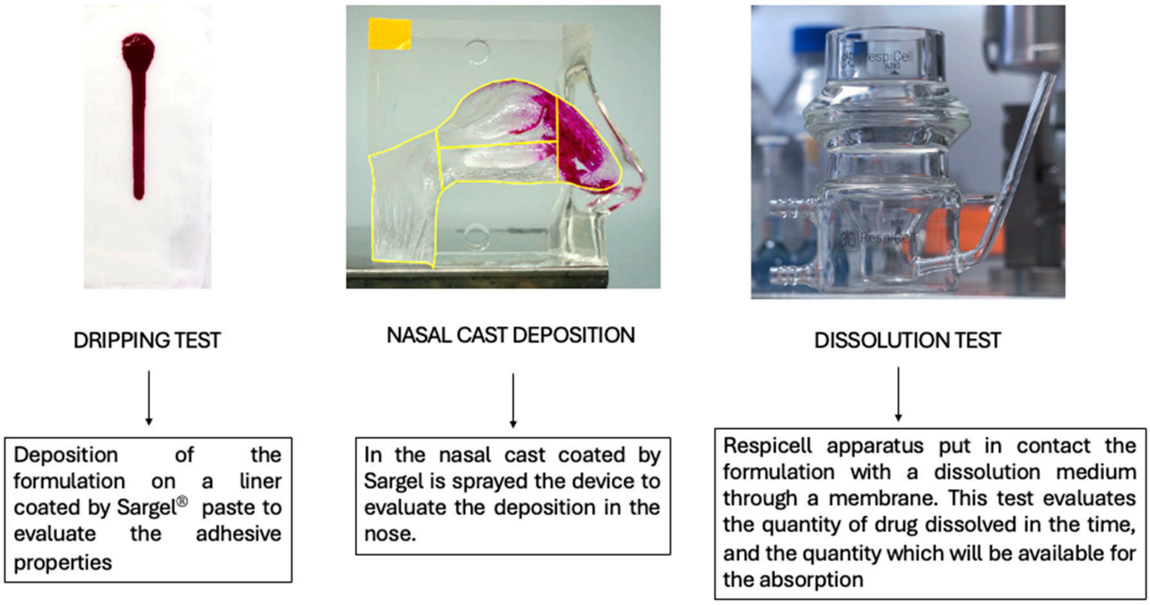

***Figure S12:** advanced tests for the characterisation of nasal spray and their effectiveness.*
